# From Risk to Balance: A Novel Approach Integrating Donor, Recipient, and Procedural Factors to Predict 12-Month Graft Loss in Liver Transplantation

**DOI:** 10.3390/jcm15114152

**Published:** 2026-05-28

**Authors:** Quirino Lai, Vibor Sesa, Fabio Melandro, Hrvoje Silovski, Robert Baronica, Stefano Ginanni Corradini, Ivona Hanzek, Gianluca Mennini, Borna Cutic, Massimo Rossi, Anna Mrzljak

**Affiliations:** 1 General Surgery and Organ Transplantation Unit, Department of General and Specialty Surgery, Azienda Ospedaliero-Universitaria Policlinico Umberto I, Sapienza University of Rome, 00161 Rome, Italyfabio.melandro@uniroma1.it (F.M.);; 2 Liver Transplant Center, University Hospital Center Zagreb, 10000 Zagreb, Croatiarobert.baronica@kbc-zagreb.hr (R.B.); anna.mrzljak@kbc-zagreb.hr (A.M.); 3 Department of Translational and Precision Medicine, Azienda Ospedaliero-Universitaria Policlinico Umberto I, Sapienza University of Rome, 00161 Rome, Italy; 4 School of Medicine, University of Zagreb, 10000 Zagreb, Croatia

**Keywords:** liver transplantation, graft loss prediction, risk stratification, ischemia time, pre-transplant assessment

## Abstract

**Background:** Early graft loss after liver transplantation (LT) remains a major challenge. Most predictive models rely on isolated factors or post-transplant variables, limiting their utility in pre-transplant decision-making. We developed a novel score integrating donor, recipient, and procedural variables to estimate 12-month graft loss based on the balance between risk and mitigation factors. **Methods:** In this retrospective study, 268 adult primary LT recipients from two European centers were analyzed. The primary endpoint was 12-month graft loss (death or retransplantation). Independent predictors were identified using multivariable logistic regression based on pre-transplant variables. A population-centered approach quantified individual deviations from average risk, classifying variables as risk exposure or mitigation factors. These were combined into a Risk-Mitigation Balance score. Model performance was evaluated using AUC and Brier score, and patients were stratified into three risk groups. **Results:** Twelve-month graft loss occurred in 17.9% of patients. Acute liver failure and warm ischemia time were the strongest predictors. The Risk-Mitigation Balance score showed good discrimination (AUC = 0.74) and calibration (Brier score 0.116), outperforming early post-transplant models (AUC = 0.65). Stratification identified three groups with significantly different graft loss rates: 7.4% (mitigation), 14.8% (intermediate), and 44.2% (risk) (*p* < 0.001), with clear separation on survival analysis. **Conclusions:** This novel score enables pre-transplant estimation of graft loss by integrating risk and modifiable factors, supporting more informed and personalized allocation decisions.

## 1. Introduction

Liver transplantation (LT) remains the only curative treatment for end-stage liver disease and selected malignancies [1]. However, early graft loss continues to represent a major clinical challenge, with significant implications for patient survival, organ allocation, and healthcare resources [2].

Several predictive models have been developed to estimate post-transplant outcomes, focusing on donor characteristics, recipient condition, or perioperative factors [3,4,5,6,7,8,9].

These models have contributed to improving risk stratification and clinical decision-making. Notably, composite indices such as the D-MELD have attempted to integrate donor and recipient variables, introducing the concept of risk balancing between the two components of the transplant process [10]. Nonetheless, most existing models rely either on isolated domains or on early post-transplant parameters, such as early allograft dysfunction (EAD) or composite scores derived from postoperative laboratory trends, which are primarily useful for guiding decisions on early retransplantation rather than pre-transplant allocation [2,6,7,11].

The ability to estimate graft loss risk exclusively based on pre-transplant variables remains a major unmet need. In clinical practice, transplant teams are required to make rapid decisions regarding donor–recipient matching under conditions of uncertainty, often integrating multiple and partially modifiable factors. These include not only baseline donor and recipient characteristics but also procedural and strategic elements such as anticipated cold ischemia time [12], expected surgical complexity and warm ischemia time [13], and the potential use of organ preservation strategies such as machine perfusion [14]. Importantly, some of these variables may act as risk mitigators, allowing clinicians to compensate for otherwise unfavorable conditions.

Current predictive tools do not adequately account for this dynamic interplay between risk and mitigation, not providing a quantitative framework to simultaneously capture the burden of risk factors and the potential impact of modifiable strategies that may offset such risk. As a result, decision-making often relies on subjective clinical judgment rather than on reproducible and transparent metrics [15,16,17].

In this context, we aimed to develop a novel pre-transplant score designed to quantify the balance between risk and mitigation factors in LT. The proposed model integrates donor, recipient, and procedural variables available before transplantation, providing an individualized estimate of the risk of graft loss and supporting more informed and consistent allocation decisions.

## 2. Materials and Methods

### 2.1. Study Design

This retrospective observational study was conducted using data from two European LT centers. The study included adult patients undergoing primary LT. Institutional Review Board approval was obtained from both participating centers, and the study was conducted in accordance with the Strengthening the Reporting of Observational Studies in Epidemiology (STROBE) guidelines.

### 2.2. Setting and Population

The study included patients from Sapienza University of Rome (AOU Policlinico Umberto I, Rome, Italy) and Zagreb University Hospital Center (Zagreb, Croatia). In Rome, 178 liver transplants were performed between 1 January 2013 and 30 April 2021, while in Zagreb, 90 liver transplants were performed between 1 May 2022 and 31 December 2025.

In the present series, no cases of retransplantation, pediatric LT (<16 years), LT with grafts reconditioned with perfusion machines, or LT with grafts from deceased cardiac donors were present.

### 2.3. Outcome

The primary outcome of the study was 12-month graft loss, defined as patient death or need for retransplantation within 12 months after transplantation. Follow-up was updated until 1 March 2026, ensuring complete outcome ascertainment of the primary endpoint.

### 2.4. Data Collection

Clinical, donor, and procedural variables were retrospectively collected from institutional databases. Data quality was ensured through internal validation procedures, including cross-checking and resolution of inconsistencies by the study investigators.

### 2.5. Definitions

Graft loss was defined as either patient death or retransplantation. Acute liver failure (ALF) referred to patients undergoing urgent LT for fulminant hepatic failure according to standard clinical criteria. Patients with acute-on-chronic liver failure (ACLF) were not classified as ALF unless they fulfilled criteria for fulminant hepatic failure requiring emergency transplantation. Early allograft dysfunction (EAD) was defined according to Olthoff criteria, while the Model for Early Allograft Function (MEAF) score was calculated using the original published formula. Organ allocation followed national and regional allocation policies, according to Centro Nazionale Trapianti (Italy) and Eurotransplant (Croatia).

### 2.6. Surgical Procedures

Liver transplantation was performed by experienced surgical teams at both centers using a standardized piggyback technique, preserving the recipient vena cava, followed by portal, arterial, and biliary reconstruction. Although minor variations may have occurred over time, the overall surgical approach was consistent within each center.

### 2.7. Statistical Analysis

Continuous variables were expressed as median and first–third quartile (Q1–Q3) and compared using the Kruskal–Wallis test. Categorical variables were reported as counts and percentages and compared using the chi-square or Fisher’s exact test, as appropriate. No missing data were observed for the variables explored in the analysis.

To identify predictors of 12-month graft loss, a multivariable logistic regression model was developed including pre-transplant donor-, recipient-, and procedure-related variables. Variable selection was performed using a backward stepwise approach, using clinically relevant covariates and those with a *p*-value ≤0.20 for constructing the model. Regression coefficients (β), standard errors (SE), odds ratios (OR), and 95% confidence intervals (95% CI) were reported.

To move beyond traditional risk prediction, a population-centered modeling approach was implemented to quantify, at the individual level, the balance between risk exposure and risk mitigation. For each variable included in the final model, individual contributions were calculated by centering the variable around its population mean (for continuous variables) or prevalence (for binary variables), and weighting this deviation by the corresponding regression coefficient (β), as follows:

Continuous variables: βj × (Xij − mean(Xj))

Binary variables: βj × (Xij − pj)

where Xij represents the value of variable j for individual i, mean(Xj) the population mean, and pj the prevalence of the binary variable. This transformation generated a centered linear predictor representing the individual deviation from the average population risk profile.

Variables were then categorized into risk exposure factors, defined as variables associated with increased probability of graft loss (positive β coefficients), and risk mitigation factors, defined as variables associated with reduced probability of graft loss (negative β coefficients). For each patient, a Risk-Mitigation Balance score was calculated as the difference between cumulative exposure and mitigation components. Model performance was assessed in terms of discrimination using the area under the receiver operating characteristic curve (AUC), and calibration using the Brier score.

Patients were subsequently stratified into three groups (i.e., mitigation, intermediate, and risk) based on the distribution of the balance score. The adopted cutoffs of the score corresponding to −0.5 and 0.5 were pragmatically selected to enhance clinical usability and interpretability.

Kaplan–Meier survival analysis and log-rank tests were used to compare graft survival across groups. A two-sided *p*-value < 0.05 was considered statistically significant. SPSS version 27.0 (IBM Corp., Armonk, NY, USA) and R software (version 4.5.0, R Foundation for Statistical Computing, Vienna, Austria) were used for analysis.

## 3. Results

A total of 268 liver transplant recipients were included in the analysis. The cohort showed a median follow-up of 56 months (Q1–Q3 =12–117). During the entire follow-up period, a total of 63 (23.5%) graft losses were reported, with 35 (13.1%) cases observed during the first 90 days and 48 (17.9%) during the first year after LT.

### 3.1. Creation and Performance Evaluation of the Risk-Mitigation Balance Score

A multivariable logistic regression model was fitted to identify independent predictors of 12-month graft loss. Acute liver failure (β = 2.39, *p* < 0.001) and warm ischemia time (β = 0.03 per minute, *p* = 0.003) emerged as the strongest contributors, while MELD score, cold ischemia time, and local donor sharing showed smaller effects but were retained in the model due to their clinical relevance and contribution to the overall risk profile (Table 1). Variables with positive regression coefficients, including acute liver failure, ischemia times, and MELD, were classified as risk exposure factors, whereas local donor sharing, characterized by a negative coefficient, was considered a mitigation factor. By applying a population-centered transformation, individual deviations from the average population profile were quantified and combined into a Risk-Mitigation Balance score, representing the net balance between factors increasing and reducing graft loss risk.

Exploring the performance of the score, the Risk-Mitigation Balance score showed the best predictive performance for 12-month graft loss, with an AUC of 0.74 (95% CI 0.66–0.83, *p* < 0.001) and a Brier score of 0.116. Its discriminative ability was superior not only to early post-transplant scores such as EAD (AUC = 0.65, 95% CI 0.56–0.74, *p* = 0.002; Brier score 0.138) and MEAF (AUC = 0.65, 95% CI 0.56–0.74, *p* = 0.003; Brier score 0.141), but also to conventional pre-transplant allocation and risk stratification models. Specifically, MELD showed an AUC of 0.67 (95% CI 0.58–0.76, *p* < 0.001; Brier score 0.138), D-MELD an AUC of 0.66 (95% CI 0.57–0.75, *p* < 0.001; Brier score 0.141), MELDNa an AUC of 0.65 (95% CI 0.56–0.74, *p* = 0.001; Brier score 0.141), GEMA-Na an AUC of 0.64 (95% CI 0.55–0.73, *p* = 0.003; Brier score 0.142), and BAR an AUC of 0.63 (95% CI 0.54–0.72, *p* = 0.005; Brier score 0.142) (Table 1).

**Figure 1:**
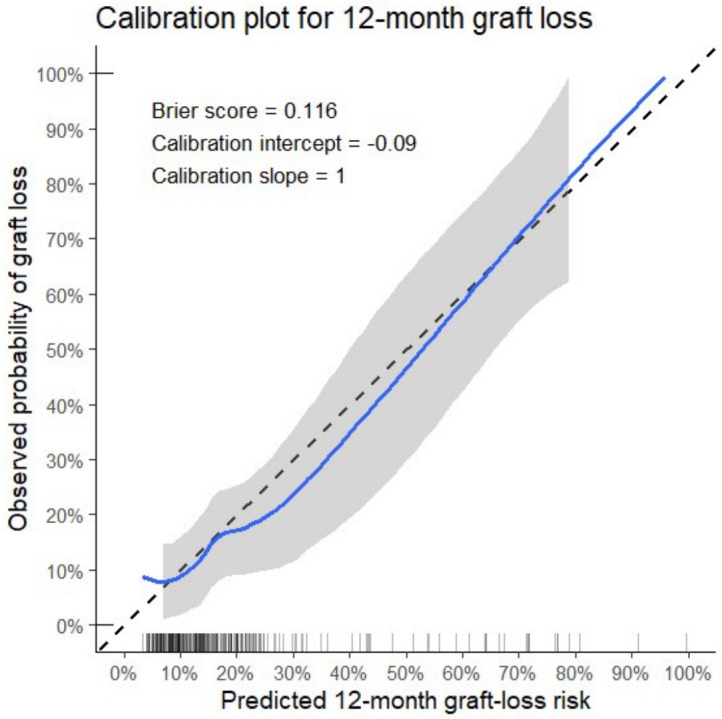
Contour plot of time-dependent transplant benefit erosion according to waiting time and non-transplant survival assumptions.

Calibration analysis further supported the reliability of the Risk-Mitigation Balance model. The calibration plot comparing predicted and observed 12-month graft-loss risk showed a good overall agreement between expected and observed probabilities across the range of predicted risk. The smoothed calibration curve remained close to the ideal reference line, with only a mild tendency toward underestimation in the highest-risk patients. This finding was consistent with the acceptable Brier score (0.116) and confirmed that the model provided not only satisfactory discrimination but also adequate calibration for clinical application (Figure 1).

Sensitivity analyses confirmed the robustness of the Risk-Mitigation Balance score across both center- and time-related subgroups. The score maintained good discriminative ability in both centers, with an AUC of 0.75 (95% CI 0.65–0.85, *p* < 0.001) in Rome and 0.70 (95% CI 0.55–0.86, *p* = 0.011) in Zagreb. Similarly, stable predictive performance was observed across different transplant eras, with an AUC of 0.76 (95% CI 0.65–0.87, *p* < 0.001) in the 2013–2019 cohort and 0.70 (95% CI 0.56–0.84, *p* = 0.006) in the 2020–2025 cohort. Brier scores remained consistently low across all subgroups (range 0.110–0.119), further supporting the reproducibility and external validity of the model (Table 1). In addition, stratification according to baseline renal function showed preserved model performance in both patients with preserved renal function (eGFR ≥60 mL/min/1.73 m^2^; AUC 0.71, 95% CI 0.60–0.82, *p* < 0.001) and those with impaired renal function (eGFR < 60 mL/min/1.73 m^2^; AUC 0.82, 95% CI 0.67–0.96, *p* < 0.001), suggesting that the score remains reliable irrespective of pre-transplant kidney function, with even stronger discrimination in higher-risk patients with renal impairment.

An additional sensitivity analysis according to ALF status confirmed preserved model performance in both ALF and non-ALF recipients. Although confidence intervals were wider in ALF recipients due to the limited sample size, the model maintained acceptable discrimination in this subgroup (AUC 0.73, 95% CI 0.52–0.94). Importantly, the score also remained significantly predictive in non-ALF recipients (AUC 0.66, 95% CI 0.56–0.77), suggesting that its predictive performance was not exclusively driven by the intrinsically high-risk profile of ALF patients.

**Figure 2:**
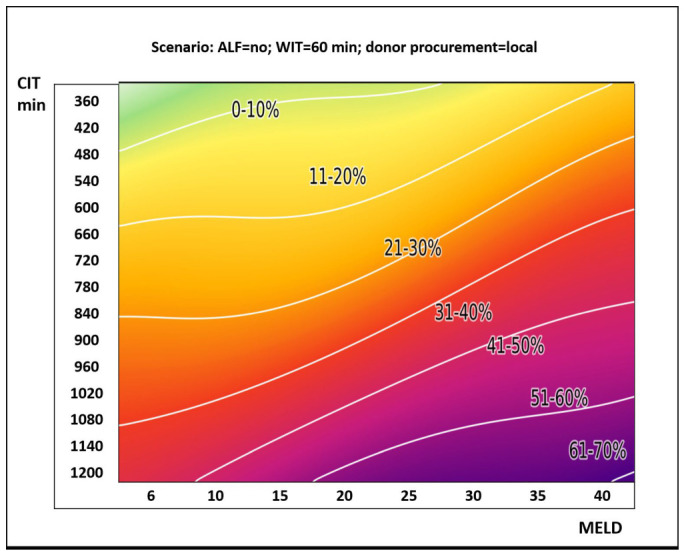
Relationship between the Risk-Mitigation Balance score and predicted probability of 12-month graft loss.

Representative clinical scenarios illustrating the clinical applicability of the Risk-Mitigation Balance score are shown in the last part of Table 1. These examples highlight how different combinations of recipient severity and procedural factors translate into markedly different predicted risks of 12-month graft loss. Patients with favorable profiles (e.g., absence of acute liver failure, lower MELD, shorter ischemia times, and local donor sharing) exhibited negative scores and negligible predicted risk. In contrast, the accumulation of adverse factors led to a progressive increase in the score and the emergence of clinically relevant risk. Importantly, the presence of acute liver failure in combination with higher MELD and unfavorable procedural characteristics resulted in a sharp increase in predicted graft loss probability, suggesting a non-linear amplification of risk when multiple adverse factors coexist (Figure 2).

### 3.2. Stratification of the Cohort in Risk Classes

Patients were stratified into three groups according to the Risk-Mitigation Balance score, namely mitigation (*n* = 94, 35.1%), intermediate (*n* = 122, 45.5%), and risk (*n* = 52, 19.4%). Baseline characteristics across the three groups are reported in Table 2. Overall, a progressive worsening of the recipient’s clinical status was observed moving from the mitigation to the risk group. MELD and MELDNa values increased significantly across groups (both *p* < 0.001), while acute liver failure was exclusively observed in the risk group (42.3%, *p* < 0.001). Waiting time on the transplant list was markedly shorter in higher-risk patients (*p* < 0.001), reflecting the urgency-driven allocation in this subgroup. Conversely, hepatocellular carcinoma was significantly more frequent in the mitigation group compared with the risk group (58.5% vs. 11.5%, *p* < 0.001), suggesting a distinct clinical and allocation profile.

Renal function showed a progressive worsening across the three groups. Although median eGFR values were lower in Group 3 compared with Groups 1 and 2 (79.5 vs. 86.4 and 85.4 mL/min, respectively), this difference did not reach statistical significance when analyzed as a continuous variable (*p* = 0.22). However, when patients were stratified according to clinically relevant renal function categories (normal ≥ 60, moderate 30–59, severe < 30 mL/min), a significant difference emerged (*p* = 0.04). In particular, Group 3 showed a higher proportion of patients with moderate-to-severe renal impairment (36.5%) compared with Group 2 (20.5%) and Group 1 (16.0%), suggesting that categorical stratification better captured the clinically meaningful deterioration in renal reserve across groups.

Donor characteristics were largely comparable across groups, with no significant differences in age, sex, or body mass index. In contrast, transplant-related variables showed a clear gradient, with both cold ischemia time and warm ischemia time progressively increasing from the mitigation to the risk group (both *p* < 0.001), supporting the role of procedural factors in modulating overall transplant risk.

Early postoperative outcomes showed a consistent and progressive deterioration across the three groups (Table 3). Patients in the risk group exhibited significantly worse biochemical markers of graft function, with higher bilirubin levels both at postoperative day 3 and day 7 (both *p* < 0.001), as well as higher MEAF scores (median 4.7 vs. 3.4 in the mitigation group, *p* < 0.001). The proportion of patients with MEAF >5 increased markedly across groups, reaching 48.1% in the risk group compared with 14.9% in the mitigation group (*p* < 0.001). Similarly, lactate dynamics reflected a worsening graft performance, with significantly higher peak lactate values observed in the risk group (*p* < 0.001). These differences translated into a significantly higher burden of postoperative complications. Major complications (Clavien-Dindo ≥ 3A) were observed in 65.4% of patients in the risk group compared with 26.6% in the mitigation group (*p* < 0.001). In parallel, both the intensive care unit stay and total hospital stay progressively increased across groups (both *p* < 0.001), further supporting the clinical relevance of the proposed stratification.

A strong and clinically meaningful gradient in graft loss was observed across the three groups. At 90 days, graft loss occurred in 5.3% of patients in the mitigation group, 9.0% in the intermediate group, and 36.5% in the risk group (*p* < 0.001). This pattern persisted at 12 months, with graft loss rates of 7.4%, 14.8%, and 44.2%, respectively (*p* < 0.001).

### 3.3. Survival Curves

**Figure 3:**
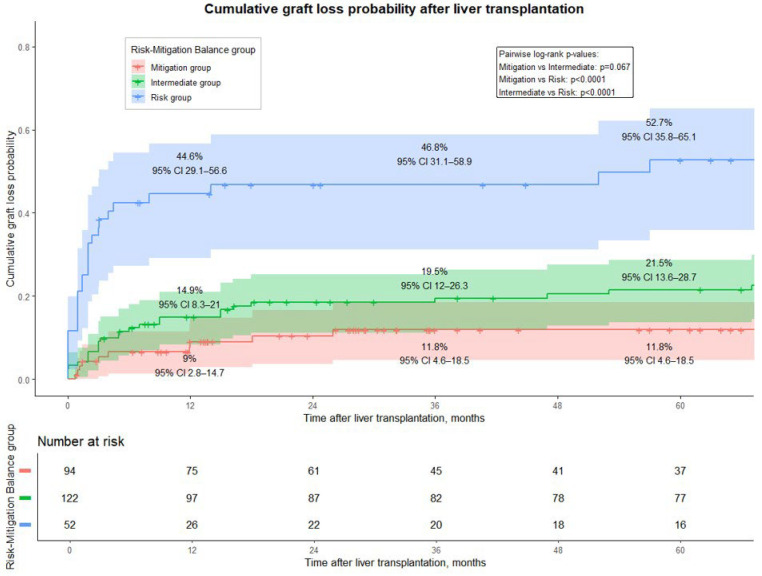
Kaplan–Meier analysis of graft loss according to Risk-Mitigation Balance score groups.

Kaplan–Meier analysis demonstrated a clear separation of graft loss curves across the three Risk-Mitigation Balance score groups (Figure 3). Patients in the mitigation group showed the lowest graft loss, followed by the intermediate group, while the risk group exhibited a markedly increased loss probability over time.

At 12, 36, and 60 months after LT, cumulative graft loss increased stepwise across the three groups, reaching 9.0% (95% CI, 2.8–14.7), 11.8% (95% CI, 4.6–18.5), and 11.8% (95% CI, 4.6–18.5) in the mitigation group; 14.9% (95% CI, 8.3–21.0), 19.5% (95% CI, 12.0–26.3), and 21.5% (95% CI, 13.6–28.7) in the intermediate group; and 44.6% (95% CI, 29.1–56.6), 46.8% (95% CI, 31.1–58.9), and 52.7% (95% CI, 35.8–65.1) in the risk group, respectively.

The differences between groups were statistically significant according to the log-rank test. Pairwise comparisons showed a significant difference between the mitigation and risk groups (*p* < 0.001), as well as between the intermediate and risk groups (*p* < 0.001), while the comparison between the mitigation and intermediate groups showed a trend toward statistical significance (*p* = 0.07).

## 4. Discussion

The present study introduces a novel conceptual and analytical framework for pre-transplant risk assessment in LT, based on the integration of donor-, recipient-, and procedure-related variables into a unified Risk-Mitigation Balance score. Unlike traditional models that primarily quantify risk, this approach explicitly incorporates the counterbalancing effect of modifiable factors, providing a more comprehensive representation of the transplant process. The score demonstrated good discriminative ability (AUC = 0.74) and outperformed established early post-transplant metrics such as EAD and MEAF, which are intrinsically limited by their retrospective nature and restricted applicability to post-transplant decision-making. Notably, this performance was achieved using exclusively pre-transplant variables, which would theoretically be expected to yield lower discriminative power compared to models incorporating post-transplant information. This further underscores the robustness and potential clinical utility of the proposed approach in pre-transplant risk stratification and decision-making.

A key finding of this study is the differential contribution of individual variables to graft loss risk. ALF emerged as the strongest determinant, with a markedly high regression coefficient, reflecting the well-known instability and high-risk profile of this patient population. Several studies have already demonstrated the higher risk of graft loss and patient death in recipients transplanted for acute or acute-on-chronic diseases [18,19]. As an example, an analysis of 4903 LT recipients with ALF from the European Liver Transplant Registry demonstrated that, despite significant improvements over time, early post-transplant mortality remained substantial. Overall patient survival was 74% at 1 year and 63% at 10 years, with inferior results with respect to all the other primary indications for LT. Notably, the combination of older recipients (>50 years) and older donors (>60 years) was associated with a strikingly high risk, with up to 57% mortality or graft loss within the first year [18]. This finding reinforces the concept that urgency-driven allocation, while necessary, inherently carries a significant biological risk that may not be fully mitigated by procedural optimization. However, the extent to which this intrinsic risk can be modified, even partially, remains uncertain and warrants further investigation [20].

Importantly, because of its strong prognostic impact, the variable “ALF” contributed substantially to the discriminative performance of the present model. However, sensitivity analyses stratified according to ALF status demonstrated preserved predictive performance even among non-ALF recipients, suggesting that the overall accuracy of the score was not solely driven by the inclusion of ALF as a dominant high-risk variable. Conversely, the wider confidence intervals and higher Brier score observed in ALF recipients likely reflect the profound clinical instability and biological heterogeneity characterizing this population.

An additional aspect deserving consideration relates to the composite definition of graft loss adopted in the present study, which combined patient death and retransplantation. Particularly in ALF recipients, mortality may reflect not only graft-related dysfunction, but also the severe systemic derangement frequently accompanying fulminant liver failure, including multiorgan failure, vasoplegia, sepsis, hemodynamic instability, and neurologic complications. Therefore, the possibility that part of the model discrimination reflects recipient-related mortality rather than isolated graft-specific dysfunction cannot be completely excluded. Nevertheless, this composite endpoint was intentionally selected to reflect clinically meaningful transplant failure in real-world practice, where graft and patient outcomes are intrinsically interconnected.

Warm ischemia time also showed a significant and independent impact. An experimental study from China investigated the impact of recipient warm ischemia time on outcomes in extended-criteria donor LT using a rat autologous orthotopic model. The results consistently showed that shorter warm ischemia time (≤10 min) was associated with significantly improved graft perfusion dynamics, better liver function, lower histological injury, and higher 3-day survival rates compared to prolonged times [21]. This large retrospective US study confirmed these results in a clinical setting of 1256 LT performed between 2000 and 2019. Progressively longer warm ischemia times are associated with increased intraoperative transfusion requirements, higher blood loss, and elevated lactate levels. Importantly, a time ≤ 30 min was independently associated with the lowest risk of early allograft dysfunction and significantly improved graft survival at both 1 and 5 years after adjustment for confounders [22].

As for the cold ischemia time, numerous articles have already explored its relevance. A large registry-based analysis including 40,288 liver transplant recipients, prolonged cold ischemia time was associated with a significant increase in graft loss, particularly within the first post-transplant year. Each additional hour of CIT conferred a 3.4% increase in the risk of graft loss (HR 1.034; *p* < 0.001). Importantly, the impact of cold ischemia time was not uniform across indications, with HCV-related cirrhosis being particularly vulnerable. In contrast, hepatocellular carcinoma or alcoholic cirrhosis demonstrated greater tolerance, with no significant impairment in graft survival up to approximately 10–12 h, and up to 13 h in patients with hepatocellular cancer and MELD score <20 [23]. These findings indicate that the detrimental effect of cold ischemia time is highly context-dependent and may be partially mitigated through tailored donor–recipient matching, allocating grafts with longer ischemia times to more resilient recipient profiles.

In a large single-center Italian study including 1354 liver transplants, the impact of cold ischemia time was further explored in the peculiar setting of aged (≥70 years) donors. Notably, the use of elderly donors was not associated with worse outcomes, with comparable rates of early retransplantation, vascular complications, and biliary complications. However, multivariable analysis identified cold ischemia time as an independent predictor of graft loss (HR = 1.0; *p* = 0.042), alongside donor age, HCV-positivity, and donor diabetes. These findings indicate that while advanced donor age alone does not necessarily impair outcomes, its impact becomes clinically relevant in the presence of additional risk factors, like particularly prolonged cold ischemia time, further highlighting the importance of optimizing modifiable procedural variables to mitigate graft-related risk [24]. Such a result is in line with our findings, in which the ages of the donor and recipient did not result in relevant parameters for the prediction of risk [25].

As for the MELD score, several studies integrated it into the risk factors for poor post-LT outcomes.

Established risk scores such as the SOFT and BAR scores incorporate recipient severity through variables such as MELD and combine multiple donor and recipient factors to predict post-transplant outcomes [8,9]. As already reported, the MELD was also combined with the donor age, generating the D-MELD. In a large national Italian study including 5946 liver transplants, the D-MELD score emerged as a strong predictor of post-transplant survival. Patients were stratified into three D-MELD classes (A <338, B 338–1628, C >1628), showing a clear risk gradient: at 3 years, recipients in class C had a significantly higher risk of death compared to class B (OR = 2.03; 95% CI = 1.44–2.85), whereas class A was protective (OR = 0.40; 95% CI = 0.24–0.66) [10].

Finally, local versus national/extranational donor sharing emerged as a protective factor, supporting the concept that logistical optimization and reduced ischemia burden can act as effective risk mitigators. This aspect has been highlighted in both European and North American series. In a Eurotransplant registry analysis including 5939 liver transplants, a region-specific donor risk model identified rescue allocation as an independent predictor of post-transplant outcomes. Notably, rescue allocation, defined as allocation after multiple prior declines due to perceived poor organ quality, often entails broader geographic sharing, and consequently longer transport times and ischemia exposure, providing a plausible mechanistic link between allocation dynamics and graft outcomes [26]. A simulation study from the United States evaluated alternative liver allocation strategies based on concentric “neighborhoods” of donor service areas, aiming to reduce geographic disparity while limiting transport burden and long ischemia times. A 500-mile model combined with existing MELD-driven policies achieved meaningful reductions in disparity and airplane travel distance while maintaining transplant activity [27]. These findings suggest that allocation policies incorporating geographically constrained sharing can improve outcomes not only by enhancing equity but also by reducing logistical complexity and ischemia exposure, highlighting the role of allocation design as a potential modifiable factor influencing transplant outcomes.

The central innovation of this study lies in shifting from a purely risk-based paradigm to a balance-based model, in which adverse and protective factors are quantified simultaneously. This approach reflects real-world clinical decision-making, where transplant teams continuously weigh unfavorable donor- and recipient-related characteristics against potentially modifiable procedural strategies. By centering each variable around its population mean and weighting it by its regression coefficient, the model captures not only the presence of risk factors but also their relative deviation from an “average” transplant scenario.

Importantly, the results suggest that graft loss risk is not simply additive but may exhibit non-linear amplification when multiple adverse factors coexist, as observed in patients combining acute liver failure, high MELD, site of graft procurement, and prolonged ischemia times.

The proposed score has several potential clinical applications. First, it may support pre-transplant decision-making, providing an objective and reproducible estimate of graft loss risk based exclusively on variables available before transplantation. This is particularly relevant in time-sensitive scenarios, where rapid yet informed allocation decisions are required. Second, the model enables scenario simulation, allowing clinicians to explore how modifications in procedural variables (e.g., reduction in cold ischemia time or optimization of surgical strategy) may influence the overall risk profile. Third, the score may serve as a tool for benchmarking and quality assessment, facilitating comparisons across centers and identifying areas where mitigation strategies could be strengthened.

One of the most relevant implications of this model concerns the potential role of organ preservation strategies, particularly machine perfusion. Although no grafts preserved with machine perfusion were included in the present cohort, the conceptual framework of the Risk-Mitigation Balance score naturally accommodates such interventions as risk mitigation factors. Machine perfusion has been shown to reduce ischemia–reperfusion injury and improve early graft function, particularly in marginal grafts [28]. Within the context of this model, its use could theoretically shift patients from a high-risk to an intermediate-risk profile by attenuating the impact of prolonged ischemia or suboptimal donor quality [29].

This highlights an important future direction: integrating dynamic or strategy-dependent variables into predictive models may allow not only risk estimation but also risk modulation, ultimately supporting more personalized and adaptive allocation strategies.

Several limitations should be acknowledged in the present study. First, the retrospective design introduces the potential for selection bias and unmeasured confounding, despite the use of multivariable modeling. Second, the relatively limited sample size and the inclusion of only two centers may affect the generalizability of the findings. External validation in larger and more heterogeneous populations is warranted. Third, some variables included in the model, particularly ischemia times, may be partially operator-dependent and subject to intra-center variability, which could limit reproducibility across different settings. Fourth, the absence of grafts preserved with machine perfusion or from DCD donors restricts the applicability of the model to contemporary transplant practice, where these strategies are increasingly adopted. More studies including these relevant parameters in the multivariable models are surely required. Finally, the selection of cutoffs for risk stratification was arbitrary and based on pragmatic considerations and may require refinement in future studies.

In conclusion, this study proposes a novel paradigm for pre-transplant risk assessment based on the integration of risk exposure and mitigation factors. By moving beyond traditional risk prediction toward a balance-based framework, the proposed model suggests a more realistic representation of the transplant process, potentially opening up new perspectives on personalized allocation strategies and optimization of transplant outcomes.

## Figures and Tables

**Table 1:** Multivariable logistic regression model for the risk of 12-month graft loss, formulas for the calculation of the Risk-Mitigation Balance Score, concordance and calibration of the score, and clinical scenarios.

Variables	Beta	SE	Wald	OR	95% CI		*p*
					Lower	Upper	
**ALF**	**2.39**	**0.63**	**14.52**	**10.90**	3.19	37.23	<0.001
WIT, min	0.03	0.01	8.88	1.03	1.01	1.05	0.003
MELD	0.03	0.02	2.01	1.03	0.99	1.07	0.16
CIT, min	0.003	0.00	1.87	1.00	0.99	1.01	0.17
Local donor sharing	−0.55	0.44	1.52	0.58	0.24	1.38	0.20
Costante	−5.00	1.09	21.06	0.01	-	-	<0.001
Formula for the calculation of the Risk-Mitigation Balance Score: 2.389 * (ALF (Y/N)–0.08) + 0.003 * (CIT min–378) + 0.027 * (WIT min–60)–0.546 * (local donor sharing (Y/N)–0.32) + 0.029 * (MELD–18) Formula for the estimation of the individual risk of 12-month graft loss: 1/[1 + e^(−1.708 + score)] §							
**Concordance and calibration of the score**							
**Score**		**SE**	**95% CI**		**AUC**	***p***	**Brier score**
			**Lower**	**Upper**			
Risk-Mitigation Balance		0.04	0.66	0.83	0.74	<0.001	0.116
MELD		0.05	0.58	0.76	0.67	<0.001	0.138
D-MELD		0.05	0.57	0.75	0.66	<0.001	0.141
MELDNa		0.05	0.56	0.74	0.65	0.001	0.141
EAD		0.05	0.56	0.74	0.65	0.002	0.138
MEAF		0.05	0.56	0.74	0.65	0.003	0.141
GEMA-Na		0.05	0.55	0.73	0.64	0.003	0.142
BAR		0.05	0.54	0.72	0.63	0.005	0.142
Donor age		0.05	0.48	0.67	0.57	0.12	0.143
Recipient age		0.05	0.41	0.59	0.50	0.96	0.147
**Sensitivity analysis on center-, time-, and clinical condition-related effect**							
Risk-Mitigation Balance		**SE**	**95% CI**		**AUC**	***p***	**Brier score**
			**Lower**	**Upper**			
Center effect: Rome		0.05	0.65	0.85	0.75	<0.001	0.119
Center effect: Zagreb		0.08	0.55	0.86	0.70	0.011	0.110
Time effect: 2013–2019		0.05	0.65	0.87	0.76	<0.001	0.117
Time effect: 2020–2025		0.07	0.56	0.84	0.70	0.006	0.114
eGFR effect: ≥60		0.06	0.60	0.82	0.71	<0.001	0.112
eGFR effect: <60		0.08	0.67	0.96	0.82	<0.001	0.130
ALF		0.11	0.52	0.94	0.73	0.03	0.200
Non-ALF		0.05	0.56	0.77	0.66	0.003	0.109
**Different clinical scenarios using the score**							
**Patient**	**ALF**	**MELD**	**CIT, min**	**WIT, min**	**Local donor share**	**Score**	**12-month graft loss prediction**
#1	No	15	250	45	Yes	−1.44	4.1%
#2	No	18	378	60	Yes	−0.56	9.4%
#3	No	25	450	90	No	1.21	37.9%
#4	Yes	40	378	60	No	3.01	78.6%
#5	Yes	40	450	90	No	4.04	91.1%

§ The Risk-Mitigation Balance score was constructed using centered covariates (mean-centered variables), the equivalent intercept for the score-based probability estimation became −1.708 after algebraic recalibration. This adjusted intercept ensures full mathematical equivalence between the original regression model based on raw variables and the simplified bedside score used for clinical applications. **Abbreviations:** SE, standard error; OR, odds ratio; CI, confidence interval; ALF, acute liver failure; WIT, warm ischemia time; MELD, Model for End-Stage Liver Disease; CIT, cold ischemia time; AUC, area under the receiver operating characteristic curve; EAD, early allograft dysfunction; MEAF, Model for Early Allograft Function.

**Table 2:** Baseline recipient, donor, and transplant characteristics according to Risk–Mitigation Balance score groups.

Variable	Mitigation Group (*n* = 94, 35.1%)	Intermediate Group (*n* = 122, 45.5%)	Risk Group (*n* = 52, 19.4%)	*p*
	Median (Q1–Q3) or *n* (%)			
**Recipient**				
**Male sex**	**77 (81.9)**	104 (85.2)	38 (73.1)	0.16
Caucasian ethnicity	93 (98.9)	118 (96.7)	51 (98.1)	0.75
Weight, kg	76 (69–86)	81 (65–90)	80 (65–89)	0.55
Height, cm	172 (168–178)	172 (168–179)	173 (165–177)	0.82
BMI	26 (23–29)	27 (23–29)	27 (22–29)	0.76
Waiting time, months	4.7 (2.6–7.9)	4.1 (1.1–9.3)	0.2 (0.1–2.8)	<0.001
Age, years	60 (54–64)	58 (50–63)	54 (45–63)	0.01
HCC	55 (58.5)	58 (47.5)	6 (11.5)	<0.001
HCV	23 (24.5)	35 (28.7)	3 (5.8)	0.004
HBV	11 (11.7)	20 (16.4)	6 (11.5)	0.53
HDV	0 (0.0)	6 (4.9)	0 (0.0)	0.04
Alcohol	45 (47.9)	57 (46.7)	20 (38.5)	0.52
MASLD	5 (5.3)	22 (18.0)	1 (1.9)	<0.001
ALF	0 (0.0)	0 (0.0)	22 (42.3)	<0.001
Other disease	9 (9.6)	17 (13.9)	4 (7.7)	0.40
Serum creatinine, mg/dL	1.0 (0.7–1.1)	1.0 (0.8–1.2)	1.1 (0.8–1.4)	0.09
Sodium, mEq/L	137 (134–140)	137 (133–139)	138 (133–140)	0.36
eGFR, mL/min	86.4 (75.2–105.2)	85.4 (67.1–102.5)	79.5 (55.9–99.8)	0.22
Normal (≥60)	79 (84.0%)	97 (79.5%)	33 (63.5%)	
Moderate (30–59)	14 (14.9%)	21 (17.2%)	15 (28.8%)	0.04
Severe (<30)	1 (1.1%)	4 (3.3%)	4 (7.7%)	
MELD	12 (8–17)	17 (12–23)	27 (21–35)	<0.001
MELDNA	12 (8–19)	18 (11–26)	29 (21–35)	<0.001
Donor				
Non-local sharing	33 (35.1)	33 (27.0)	19 (36.5)	0.32
Age, years	62 (50–68)	59 (47–68)	59 (48–68)	0.84
Male sex	54 (57.4)	62 (50.8)	24 (46.2)	0.39
Cause of death				
Trauma	19 (20.2)	32 (26.2)	21 (40.4)	0.03
Anoxia	3 (3.2)	5 (4.1)	1 (1.9)	0.91
CVA	68 (72.3)	86 (70.5)	29 (55.8)	0.09
Other cause	2 (2.1)	3 (2.5)	1 (1.9)	1.00
ICU stay, days	5 (3–6)	3 (3–6)	4 (2–5)	0.06
Weight, kg	75 (70–90)	73 (65–85)	74 (68–86)	0.19
Height, cm	170 (165–180)	168 (164–175)	168 (165–175)	0.14
BMI	26 (24–29)	26 (24–29)	26 (24–29)	0.70
Transplantation				
CIT	336 (236–400)	400 (376–425)	398 (351–422)	<0.001
WIT	45 (37–57)	63 (60–70)	73 (63–90)	<0.001

**Abbreviations:** BMI, body mass index; HCC, hepatocellular carcinoma; HCV, hepatitis C virus; HBV, hepatitis B virus; HDV, hepatitis D virus; MASLD, metabolic dysfunction-associated steatotic liver disease; ALF, acute liver failure; eGFR, estimated glomerular filtration rate; MELD, Model for End-Stage Liver Disease; MELDNa, Model for End-Stage Liver Disease including serum sodium; CVA, cerebrovascular accident; ICU, intensive care unit; CIT, cold ischemia time; WIT, warm ischemia time.

**Table 3:** Early postoperative outcomes and graft loss according to Risk–Mitigation Balance score groups.

Variable	Mitigation Group (*n* = 94, 35.1%)	Intermediate Group (*n* = 122, 45.5%)	Risk Group (*n* = 52, 19.4%)	*p*
	Median (Q1–Q3) or *n* (%)			
**AST IU/L peak ≤** **3 days**	**905 (595–1494)**	**820 (483–1456)**	832 (513–1545)	0.74
ALT IU/L peak ≤ 3 days	590 (283–1085)	504 (321–1110)	668 (382–1237)	0.40
Transaminases > 2000 IU/L ≤ 3 days	14 (14.9)	20 (16.4)	11 (21.2)	0.62
Bilirubin mg/dL ≤ 3 days	1.0 (0.6–3.0)	2.9 (1.8–6.0)	3.1 (1.9–6.2)	<0.001
Bilirubin mg/dL at day 7	1.5 (0.5–4.8)	6.1 (2.1–10.6)	4.8 (1.8–11.3)	<0.001
Bilirubin >10 mg/dL at day 7	10 (10.6)	35 (28.7)	13 (25.0)	0.005
INR peak ≤ 3 days	1.41 (1.28–1.61)	1.41 (1.29–1.59)	1.54 (1.38–1.77)	0.07
INR at day 7	1.12 (1.04–1.25)	1.17 (1.09–1.32)	1.17 (1.04–1.27)	0.06
INR >1.6 at day 7	3 (3.2)	12 (9.8)	3 (5.8)	0.14
EAD	21 (22.3)	43 (35.2)	20 (38.5)	0.06
MEAF	3.4 (2.3–4.4)	4.1 (2.7–5.4)	4.7 (3.3–6.1)	<0.001
MEAF >5	14 (14.9)	36 (29.5)	25 (48.1)	<0.001
Lactates mmol/L at declamping	3.7 (3.1–4.6)	3.5 (2.8–4.17)	4.1 (3.4–5.0)	0.001
Lactates mmol/L at LT end	2.0 (1.2–2.9)	2.2 (1.6–3.2)	2.8 (2.2–3.7)	<0.001
Lactates mmol/L ≤ 24 h	1.5 (0.9–2.1)	1.6 (1.1–2.2)	1.7 (1.1–3.1)	0.57
Lactates mmol/L peak ≤ 24 h	3.9 (3.1–4.9)	3.8 (2.9–4.7)	5.0 (3.9–8.4)	<0.001
Clavien Dindo ≥ 3A	25 (26.6)	40 (32.8)	34 (65.4)	<0.001
ICU length of stay, days	4 (3–7)	6 (4–12)	8 (3–16)	<0.001
Hospital length of stay, days	16 (12–26)	18 (15–32)	27 (16–40)	<0.001
Graft loss ≤ 90 days	5 (5.3)	11 (9.0)	19 (36.5)	<0.001
Graft loss ≤ 12 months	7 (7.4)	18 (14.8)	23 (44.2)	<0.001
Graft loss entire period of follow-up	10 (10.6)	27 (22.1)	26 (50.0)	<0.001
Death entire period of follow-up	10 (10.6)	26 (21.3)	24 (46.2)	<0.001
Cause:				
Cardiac	1 (1.1)	1 (0.8%)	1 (1.9%)	0.78
Neurologic	0 (-)	2 (1.6%)	0 (0.0%)	0.68
Infective	2 (2.1)	4 (3.3%)	7 (13.5%)	0.01
Biliary	2 (2.1)	2 (1.6%)	1 (1.9%)	1.00
MOF	4 (4.3)	1 (0.8%)	5 (9.6%)	0.02
PNF/PDF/DNF	0 (-)	2 (1.6%)	3 (5.8%)	0.048
Other	1 (1.1)	14 (11.5%)	7 (13.5%)	0.002

**Abbreviations:** AST, aspartate aminotransferase; ALT, alanine aminotransferase; INR, international normalized ratio; EAD, early allograft dysfunction; MEAF, Model for Early Allograft Function; LT, liver transplantation; ICU, intensive care unit; PNF, primary-non-function; PDF, primary dysfunction; DNF, delayed non-function.

## Data Availability

The original contributions presented in this study are included in the article. Further inquiries can be directed to the corresponding author.
